# Effects of Moderate Consumption of a Probiotic‐Fermented Sour Beer on the Inflammatory, Immunity, Lipid Profile, and Gut Microbiome of Healthy Men in a Participant‐Blinded, Randomized‐Controlled Within‐Subject Crossover Study

**DOI:** 10.1002/fsn3.4627

**Published:** 2024-12-01

**Authors:** Sean Jun Leong Ou, Hafizah Yusri, Dimeng Yang, Chin Meng Khoo, Mei Hui Liu

**Affiliations:** ^1^ Department of Food Science & Technology National University of Singapore Singapore Singapore; ^2^ Department of Medicine, Yong Loo Lin School of Medicine National University of Singapore Singapore Singapore

**Keywords:** beer, gut microbiome, inflammation, *Lacticaseibacillus paracasei*, lipid profile, probiotic

## Abstract

Probiotic sour beer (PRO) fermented with *Lacticaseibacillus paracasei* Lpc‐37 is a novel beverage option, which may potentially offer health benefits. In this study, the effects of PRO are evaluated on the inflammatory, immunity, lipid profile, and gut microbiome of consumers in a 5‐week, participant‐blinded, randomized‐controlled within‐subject crossover study. Twenty‐one healthy male participants consumed 330 mL of PRO and normal sour beer (CON) daily for 2 weeks each with a 1 week of washout. Stool and blood samples were collected before and after each intervention. Significant increases for *Proteobacteria* and *Bacteroides* and a significant decrease in *Dialister* (*p* < 0.05) were observed in the CON group, while gut microbiome populations remained relatively stable in the PRO group. A significant increase was also found in HDL‐cholesterol after PRO (*p* < 0.05), while no significant differences were observed in inflammatory and immunity profiles. Further research is warranted to explore its HDL‐cholesterol increasing potential.

## Graphical abstract

In this randomized crossover study, the moderate consumption of a probiotic sour beer fermented with *Lacticaseibacillus paracasei* Lpc‐37 among healthy male volunteers resulted in a significant increase in HDL‐cholesterol. It also contributed to the balance of gut microbiome composition in comparison with a control sour beer.

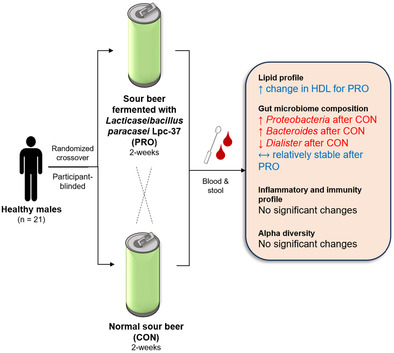

## 1 Introduction

Beer is a popular alcoholic beverage recognized as one of the culturally oldest fermented beverages in the world (Wunderlich and Back 2009) and has gained wide recognition for their various types of unique and exclusive beer styles. New innovations and technologies in brewing industries have rapidly expanded the repertoire of beers, including the development of beers with enhanced functional properties aimed at supplementing consumer health (Aquilani et al. 2015; Capece et al. 2018; Loh et al. 2021). Among traditional fermented drinks, including kombucha (fermented tea) and kefir (fermented milk), which lactic acid bacteria (LAB) have been identified as the predominant microorganism present among the consortium of other microorganisms, LAB has also recently been incorporated as probiotics into unpasteurized beer, aimed at conferring potential health benefits to consumers.

The regular intake of probiotics has been associated with several health benefits, such as improvements to gut microbiome balance, immunity (Khalesi et al. 2019), and blood lipid profiles (Gadelha and Bezerra 2019). Probiotics such as *Lacticaseibacillus* (formerly *Lactobacillus*), *Bifidobacterium*, and *Saccharomyces* strains have been widely utilized, and studies have demonstrated their potential health‐promoting properties on the gut microbiome (Sanders et al. 2019) through the production of metabolites, mainly short‐chain fatty acids (SCFAs). SCFAs such as butyrate, propionate, and acetate may be used by enterocytes for intestinal epithelial barrier function (Yoo et al. 2020) or exported into the bloodstream where they may interfere with signaling cascades through the inhibition of histone deacetylases (HDACs) and the activation of G‐protein‐coupled receptors (GPCRs) (Tan et al. 2014). Such regulatory mechanisms have been associated with modulatory effects on immune homeostasis, inflammation, and metabolism (Tan et al. 2014).

Although the use of bacteria is not foreign in the fermentation of beer, such as in the use of LAB in beer acidification processes, little is still known about the health‐promoting efficacy of using beer as a delivery vehicle for probiotics. Previous studies have investigated the probiotic viability of LAB‐fermented sour beer and the amino acid metabolites present in the beers. Chan et al. (2019) demonstrated high probiotic cell counts of *Lacticaseibacillus paracasei* ( *L. paracasei* ) L26 when cofermented with yeast in unhopped worts over a period of 10 days. This indicated the feasibility of *L. paracasei* to be supplemented through unhopped beers as a new form of craft beer. Additionally, Loh et al. (2021) reported high contents of branched‐chain and aromatic amino acid metabolites produced by *L. paracasei* Lpc‐37 and *Saccharomyces cerevisiae* CNCM I‐3856‐fermented unhopped beers using a targeted and nontargeted metabolomics approach. These findings may further ascribe LAB probiotics‐fermented beers to a high phenolic content and antioxidant capacity beneficial for consumer health.

While current studies have supported the viability of probiotics delivery through a beer medium and the potential health promoting effects from LAB probiotics intake, its clinical effects in vivo have not been elucidated. This study thus aimed to examine the effects of a 2‐week acute moderate consumption of a sour beer fermented with a probiotic strain, *L. paracasei* Lpc‐37, on inflammatory markers, immunity, lipid profile, and gut microbiome composition among healthy volunteers, in comparison with a sour beer control (CON).

## 2 Materials and Methods

### 2.1 Study Design

**FIGURE 1:**
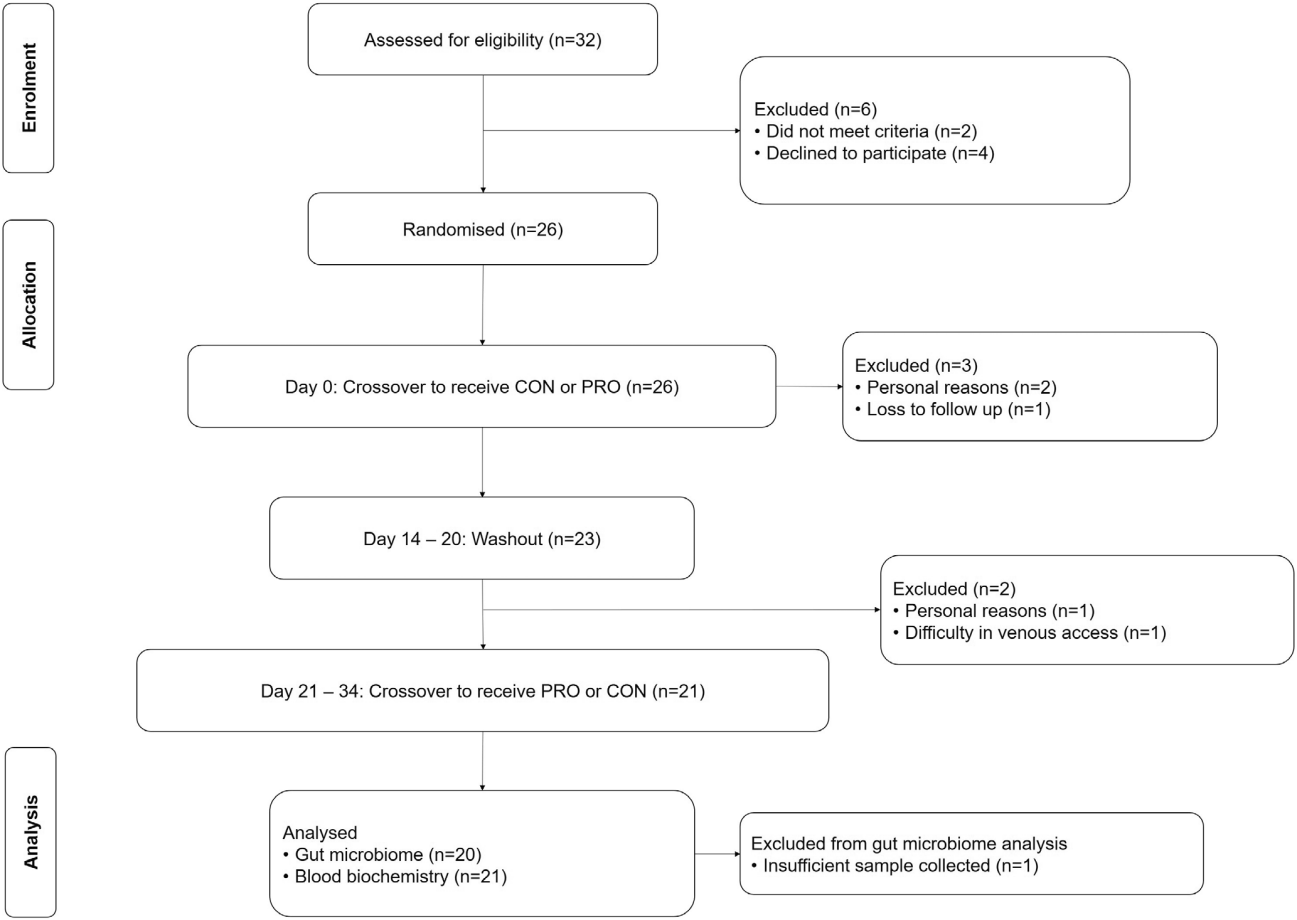
Consolidated Standards of Reporting Trials (CONSORT) diagram. All 21 participants were used for analysis, except for gut microbiome analysis involving stool samples where only 20 participants were used due to insufficient sample amount provided by a participant.

This 2 × 2 crossover participant‐blinded RCT comprised two 2‐week intervention periods, with a 1‐week washout period in between to allow the measured parameters to return to baseline (Figure 1). The 1‐week washout period was employed by referencing previous similar studies (Bautista‐Gallego et al. 2019; Sierksma et al. 2002). Participants were block‐randomized online into two sequences, in which they were instructed to consume one drink (330 mL) of CON or PRO every evening during the intervention periods. Stool samples for the characterization of gut microbiome, and fasting blood samples for the quantification of immune and biomarkers were collected on the first and last day of each intervention period. Prior to each study visit, participants were instructed to fast for 10 h, and avoid high fat foods and caffeinated beverages 24 h and 4–6 h prior to reporting for the visit, respectively. Participants were also instructed to abstain from alcohol and probiotic‐containing foods, beverages, and supplementation 1 week before the start of the study as well as during the washout. On the day of the study visit, participants were instructed to rest for 15 min in a supinated position before blood pressure measurements. Fasting blood samples were then obtained through venipuncture from the antecubital vein on the arm not used for blood pressure measurements. Stool samples were also collected using a commercial stool microbial collection and stabilization kit within 24 h before the study visit. The daily amount of alcohol consumed in the study falls within the recommended alcohol limits indicated by the national digital health platform HealthHub, Singapore (HealthHub 2023), which states that men should consume no more than two standard drinks a day (defined as one can, or 330 mL of regular beer). Compliance to the sour beer interventions was checked daily with participants notifying investigators of the unique serial number labeled on each can of sour beer consumed. The study was approved and conducted following the institutional guidelines by the National Healthcare Group Domain Specific Review Board (Ref 2021/00196), and registered at ClinicalTrials.gov (NCT05401604).

### 2.2 Participants

Twenty‐six healthy Asian Chinese men aged between 21 and 60 who were non‐naïve alcohol drinkers were recruited through posters and personal communication around the National University of Singapore campus grounds. Participants who had a history of alcoholism, smoking, were on regular medication (western or traditional), were on antibiotic prescriptions less than 2 months prior, or had medical history that may affect the interpretation of results were excluded from the study. Of the 26 participants enrolled, five dropped out due to personal reasons, loss of contact, and difficulty in venous access. The remaining 21 participants had completed the study and were included for analysis. All participants gave written informed consent prior to their study enrolment.

### 2.3 Biological Sample Collection and Measurements

Stool samples were collected using the commercial OMNIgene•GUT stool microbial collection and stabilization kit (OM‐200, DNA Genotek Inc., Ontario, Canada) for the assessment of *L. paracasei* Lpc‐37 stability in stools and the characterization of the gut microbiome. Stabilized stool samples were lyophilized and stored at −80°C prior to further gut microbiome analyses.

Phlebotomy was carried out on the antecubital vein on the arm not used for blood pressure measurements. Blood samples for white blood cell (WBC) count (total count, neutrophils, lymphocytes, monocytes, eosinophils, and basophils) measurements were collected in EDTA vacutainers and inverted six to eight times post blood collection. Blood samples for triglycerides (TG), total cholesterol (TC), HDL‐cholesterol (HDL‐c), LDL‐cholesterol (LDL‐c), and enzymes aspartate transferase (AST), alanine aminotransferase (ALT), and lactate dehydrogenase (LDH) measurements were collected in plain vacutainers and left to clot for 30 min. Additional blood samples for the assessment of cytokines were collected in EDTA vacutainers and inverted six to eight times post blood collection—plasma was separated from these samples via centrifugation at 2500 × g for 15 min at 4°C and were stored at −80°C prior to analysis. Blood samples for WBC counts, TG, TC, HDL‐c, and LDL‐c were sent to National University Hospital Referral Laboratories for biochemical analyses. WBC counts were determined by light scattering flow cytometry, TG was determined by the glycerol phosphate oxidase method, TC was determined by the cholesterol esterase/cholesterol oxidase method, HDL‐c was determined by the accelerator selective detergent method, and LDL‐c was measured by the liquid selective detergent method. Plasma separated from additional blood samples were analyzed for cytokines interferon gamma (IFN‐γ), interleukins (IL1b, 2, 4, 5, 10, 12p70), and tumor necrosis factor alpha (TNF‐α) using the ELLA automated ELISA immunoassay system (Protein Simple, California, USA) following the manufacturer's protocol.

### 2.4 Sour Beer Samples

Probiotic and control sour beer samples were codeveloped by a technical company (Probicient Pte Ltd., Singapore) and a licensed local brewery (Brewerkz Brewing Co., Singapore), according to previously patented methods by Liu and Chan (2018). As compared to PRO, the CON samples were fermented in the absence of probiotics. Other ingredients used in the production of both sour beer samples comprised water, malt, yeast, and raspberry puree for raspberry flavor and acidification. Both sour beer samples had identical packaging and were produced to have similar sensory profiles to facilitate the blinding of participants. The pH of both sour beer samples was 3.2 ± 0.2 measured using a Seven Compact pH meter S220 (Mettler Toledo, Greifensee, Switzerland). Each serving of sour beer comprised 330 mL of beer in a can with an alcohol concentration between 4.5 ± 0.2% determined using an alcohol meter (Alcolyzer, Anton Paar GmbH, Graz, Austria). Both sour beer samples were stored at 5°C prior to analyses and consumption.

Microbial enumeration was conducted on PRO to ensure the stability of *Lacticaseibacillus* throughout the duration of the study period for every 14 days. Serial dilutions were carried out in 0.1% (w/v) bacteriological peptone (Oxoid Ltd., Hampshire, England) and 0.85% (w/v) of sodium chloride (Goodrich Chemical Enterprise, Singapore). Appropriate dilutions were spread‐plated or pourplated on selective agar in duplicates. MRS agar was used and supplemented with 0.5 g/L antifungal natamycin (Natamax, Danisco A/S, Copenhagen, Denmark) to inhibit yeasts. The plates were incubated at 37°C for 48 h before counting. Microbial enumeration demonstrated decreasing stability of *Lacticaseibacillus* in PRO over the storage period of 264 days (Figure S1). From the figure, storage of PRO at 5°C for a maximum of 250 days would permit the *Lacticaseibacillus* concentration in PRO to fall from 7.77 log CFU/mL to a minimum of 6.72 log CFU/mL, which was above the minimum probiotic concentration of 6.5 log CFU/mL required to confer health benefits according to Hill et al. (2014). This ensured sufficient viability and adequate amounts of *Lacticaseibacillus* in the beer during this duration for the execution of the clinical study.

### 2.5 Gut Microbiome Profiling of Stool Samples

Stool samples were sent to an external laboratory for profiling of the gut microbiome (taxonomic groups and alpha diversity indices: chao1, observed OTUs, Shannon's index, and Simpson's index) with 16S rDNA (V3–V4 region) amplicon metagenome sequencing using the Illumina NovaSeq sequencing platform (Novogene AIT Genomics Singapore Pte, Singapore), according to previous similarly described methods (Zhou et al. 2018). Briefly, the universal primer set 341F (5′‐CCT AYG GGR BGC ASC AG‐3′) and 806R (5′‐GGA CTA CNN GGG TAT CTA AT‐3′) was used for the amplification of the V3–V4 regions of bacterial 16S rRNA. From the DNA libraries generated, paired‐end sequencing of libraries was performed on the Illumina platform, and raw reads were assigned to various samples based on their unique barcodes. Primer and barcode sequences were truncated, and raw reads were merged to form raw tags using the FLASH v1.2.11 software. Raw tags were quality‐controlled using the fastp software, and clean tags were obtained. The Vsearch software was used to blast clean tags for chimera filtering to retrieve effective tags. DADA2 in QIIME2 was used to filter out sequences from effective tags with abundance less than 5 to generate the final Amplicon Sequence Variants (ASVs). From this, the classify‐sklearn module in QIIME2 was used for comparison of ASVs and to obtain the species annotation and abundance distribution for each ASVs. The relative abundance (%) at the phylum and genus taxonomic levels were computed, and alpha diversities were measured using QIIME2.

Additionally, the presence of *L. paracasei* Lpc‐37 in stool samples before and after PRO and CON were confirmed by further sequencing analysis. DNA was extracted from stool samples using reagents and consumables by ZYMO Research *Quick*‐DNA Fecal/Soil Microbe Miniprep Kit (ZYMO Research, Irvine, California, USA) according to the manufacturer's protocol. Briefly, stool samples mixed in OMNIgene•GUT kit stabilizing solution were lysed and homogenized using a MP Biomedicals FastPrep‐24 5G sample preparation system (Bio Laboratories Pte Ltd., Singapore) at 6.0 m/s for 40 s and centrifuged at 10,000 × g for 1 min. The supernatant for each sample was collected and filtered. For DNA isolation, the filtrate was lysed upon the addition of a Genomic Lysis Buffer and transferred into a spin column. The DNA fragments captured on the spin column were rinsed and then eluted into microcentrifuge tubes. DNA concentration and purity from the extracted DNA samples were examined using BioDrop DUO+ Nanodrop Analyzer (DKSH Management Ltd., Switzerland) and stored at −80°C until further analysis.

Polymerase chain reaction (PCR) was conducted in 10 μL reaction mixtures consisting of 3.5 μL of extracted DNA sample, 5 μL Promega GoTaq PCR Master Mix (Promega Corporation, Madison, Wisconsin, USA), 1 μL nuclease‐free water, and a total of 0.5 μL of specific forward and reverse *L. paracasei* Lpc‐37 primers (Integrated DNA Technologies Pte Ltd., Singapore). The sequence of the forward primer was 5′ CCAACCACCTTGAGTTCTCGT 3′, and the sequence of the reverse primer was 5′ TCCGTTGGCAAACCCAGT 3′. PCR cycling conditions used were as follows: 1 cycle of GoTaq DNA polymerase activation (95°C) for 2 min, and a total of 40 cycles of denaturation (95°C), annealing and extension (60°C) for 15 s and 1 min, respectively, using Veriti 96‐Well Thermal Cycler (Applied Biosystems, Thermo Fisher Scientific Inc., Massachusetts, USA). PCR products were subjected to gel electrophoresis by mixing 5 μL of PCR product with 1 μL of gel loading dye and loading the mixture into 1.5% agarose gel with SYBR safe DNA gel stain. A low‐molecular‐weight DNA ladder was used with a size range of 25 bp to 766 bp (New England Biolabs Inc., Ipswich, Massachusetts, USA), and the gel was run at 100 V for 30 min. The DNA bands were visualized using iBright CL1500 Imaging System (Invitrogen, Thermo Fisher Scientific Inc., Massachusetts, USA). PCR products were cleaned up and sequenced (DNA Standard Sequencing, Bio Basic Asia Pacific Pte Ltd., Singapore) using the same primers. DNA sequences of each sample were checked against the National Institutes of Health's Basic Local Alignment Search Tool (BLAST). The presence of matched *L. paracasei* Lpc‐37 sequences for each participant was recorded (Figure S2; Tables S1 and S2).

### 2.6 Statistical Analysis

Due to the complexities of gut microbiome data involving analyses of various taxonomic classifications, diversities, and the dynamic nature of gut microbial communities, sample size calculation based on previous gut microbiome data may present a challenge. As such, sample size was estimated according to findings by Romeo et al. (2007), which found an increase in the anti‐inflammatory cytokine IL‐10 from 243.1 to 801.5 pg/mL following moderate consumption of beer. Using a power of 80% and a significance level of α = 0.05, a minimum of 21 participants were estimated to be required. To account for subject attrition, 26 participants were finally recruited for the study.

Statistical analyses were performed using GraphPad Prism 9.0 (GraphPad, San Diego, CA, USA), with statistical significance determined at *p* < 0.05. Data were checked for normality using the Shapiro–Wilk test. Statistical differences in the changes in inflammatory, immunity, lipid profiles, and gut microbiome in the CON and PRO intervention groups were analyzed using repeated measures one‐way analysis of variance (ANOVA) with Tukey's post hoc test for normally distributed data, or Friedman test with Dunn's post hoc test for non‐normally distributed data. Values are presented as (mean, 95% CI [LL, UL]), where CI, confidence intervals; LL, lower limit; UL, upper limit, unless otherwise stated.

## 3 Results

### 3.1 Baseline Characteristics

Twenty‐six participants were enrolled and randomized into the study interventions (Figure 1). Of those enrolled, 21 participants had completed the study procedures and were included for analysis. One participant had withdrawn from the study due to insufficient venous access during phlebotomy. Three participants had withdrawn from the study due to personal reasons. One participant was lost to follow‐up attempts by study investigators. None of the participants had reported any adverse events from the consumption of the sour beer samples. The baseline characteristics of the participants are summarized in Table 1.

**TABLE 1:** Baseline characteristics of participants who completed the study (*n* = 21).

	Mean ± SD	Normal range
Age, years	30 ± 10	—
BMI, kg/m^2^	23.4 ± 3.0	—
Percentage body fat, %	22.39 ± 7.80	—
Triglyceride, mmol/L	1.15 ± 0.61	1.7–2.0
Cholesterol, mmol/L	4.91 ± 0.92	< 5.2
HDL‐cholesterol, mmol/L	1.41 ± 0.25	1.0–1.5
LDL‐cholesterol, mmol/L	3.04 ± 0.94	2.6–3.3
Fasting glucose, mmol/L	4.76 ± 0.45	3–6
AST (U/L)	24.29 ± 8.14	10.0–50.0
ALT (U/L)	21.76 ± 9.51	10.0–70.0
LDH (U/L)	363.29 ± 78.96	250.0–580.0

*Note:* Values are presented as mean ± standard deviation (SD).

### 3.2 Acute Effects of Probiotic Sour Beer on Lipid Profiles

**FIGURE 2:**
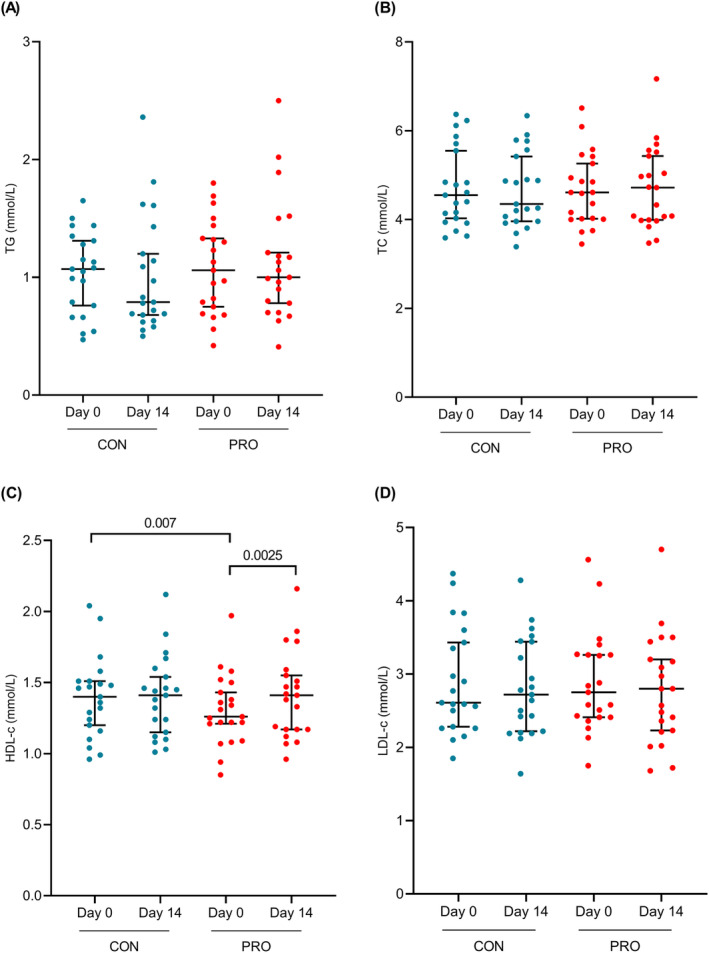
Postinterventional changes in fasting lipid panel concentrations. (A) TG, (B) TC, (C) HDL‐c, (D) LDL‐c. Graphs are presented as medians and 95% CI. Differences in postinterventional changes were analyzed with repeated measures one‐way ANOVA or Friedman test and statistical significance was determined at *p* < 0.05.

Changes in fasting plasma concentrations of lipid panel markers from baseline in response to the CON and PRO interventions are presented in Figure 2. Fasting lipid concentrations did not reflect any significant changes in response to CON or PRO interventions, except for HDL‐c. Changes in postinterventional fasting HDL‐c concentrations were significantly higher on the 14th day of PRO (1.41 mmol/L, 95% CI [1.27, 1.55], *p* = 0.0025), but this was not observed on the 14th day of CON (1.40 mmol/L, 95% CI [1.27, 1.53], *p* = 0.8308). A significant difference was also observed between the HDL‐c concentrations on the 1st day of CON and PRO (*p* = 0.007) at (1.39 mmol/L, 95% CI [1.26, 1.52]) and (1.31 mmol/L, 95% CI [1.19, 1.42]), respectively.

### 3.3 Acute Effects of Probiotic Sour Beer on Inflammatory Markers

**FIGURE 3:**
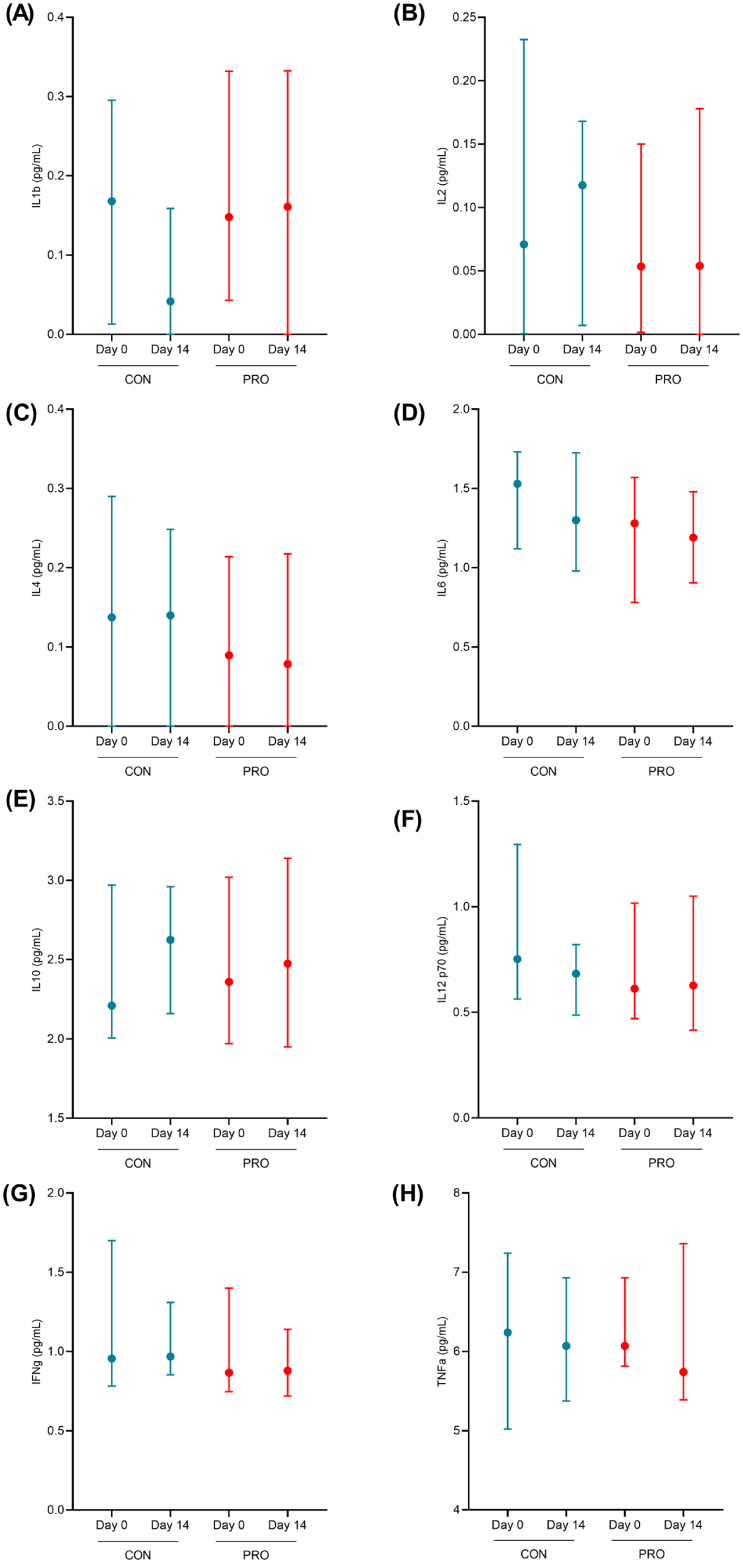
Postinterventional changes in fasting inflammatory marker concentrations. (A) IL1b, (B) IL2, (C) IL4, (D) IL6, (E) IL10, (F) IL12p70, (G) IFN‐γ, (H) TNF‐α. Graphs are presented as medians and 95% CI. Differences in post‐interventional changes were analyzed with repeated measures one‐way ANOVA or Friedman test and statistical significance was determined at *p* < 0.05.

Postinterventional changes in inflammatory profile are as shown in Figure 3. Relative to CON, no changes in fasting concentrations of inflammatory cytokines after PRO were noted.

### 3.4 Acute Effects of Probiotic Sour Beer on Immunity Markers

**FIGURE 4:**
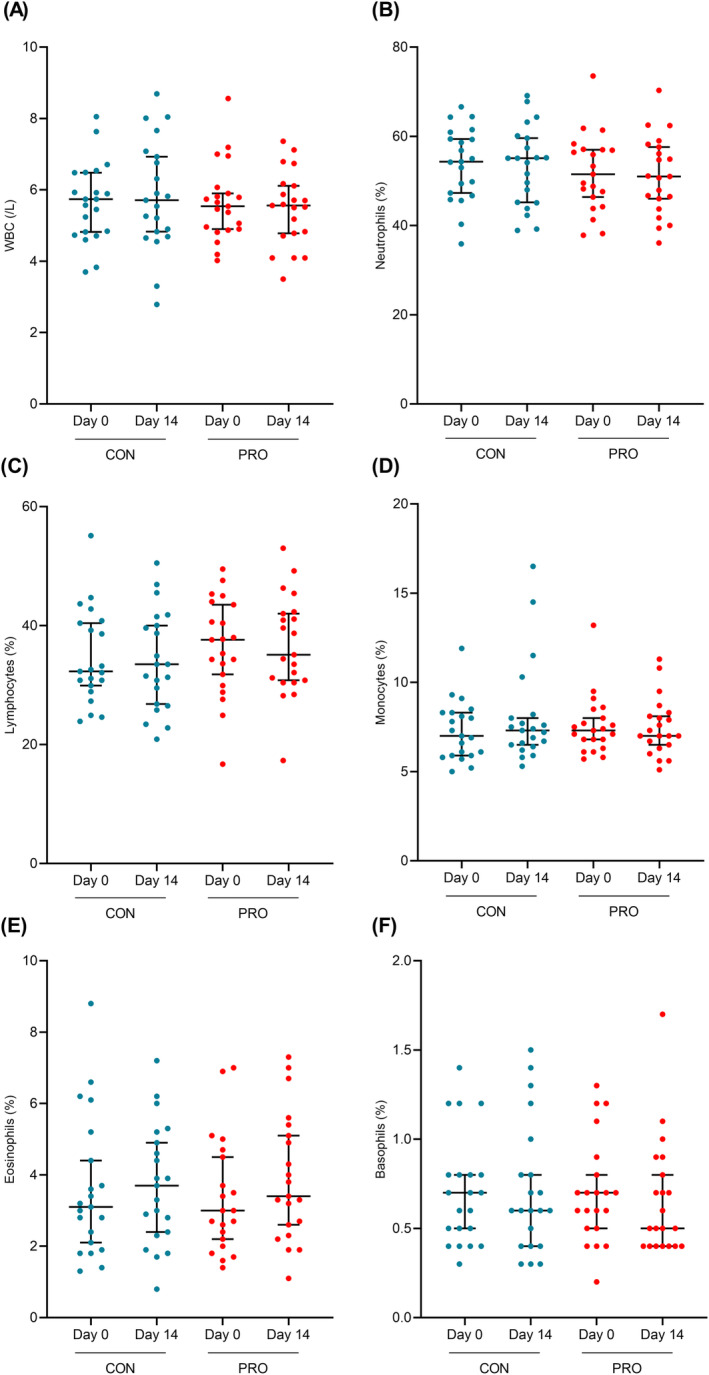
Postinterventional changes in fasting immunity marker concentrations. (A) WBC, (B) neutrophils, (C) lymphocytes, (D) monocytes, (E) eosinophils, (F) basophils. Graphs are presented as medians and 95% CI. Differences in postinterventional changes were analyzed with repeated measures one‐way ANOVA or Friedman test and statistical significance was determined at *p* < 0.05.

Differences in fasting concentrations of immunity markers between PRO and CON intervention periods were not statistically significant, as represented in Figure 4.

### 3.5 Acute Effects of Probiotic Sour Beer on Human Gut Microbiota Community

**FIGURE 5:**
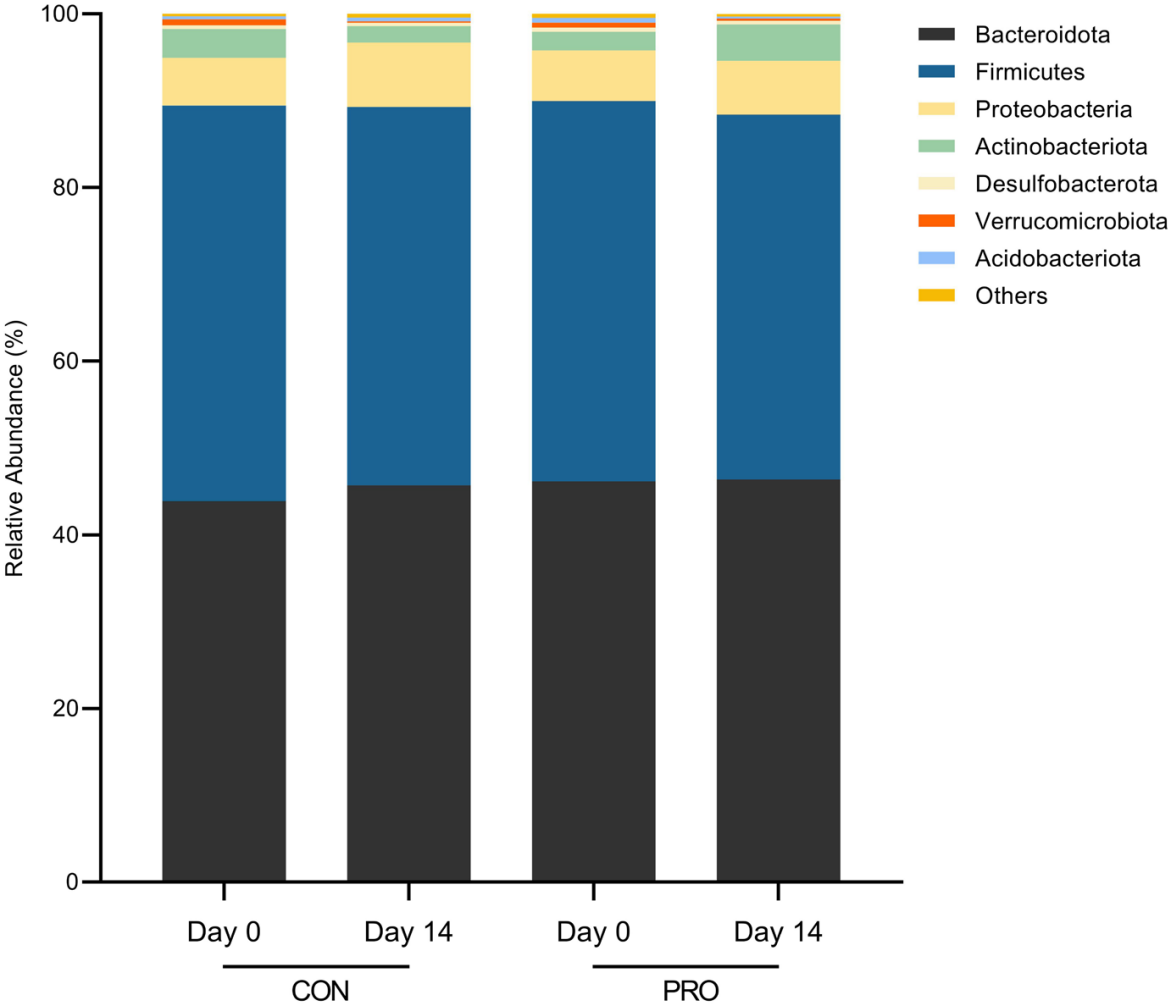
Relative abundance of gut microbiome at the phylum level before and after each intervention period (*n* = 21).

**FIGURE 6:**
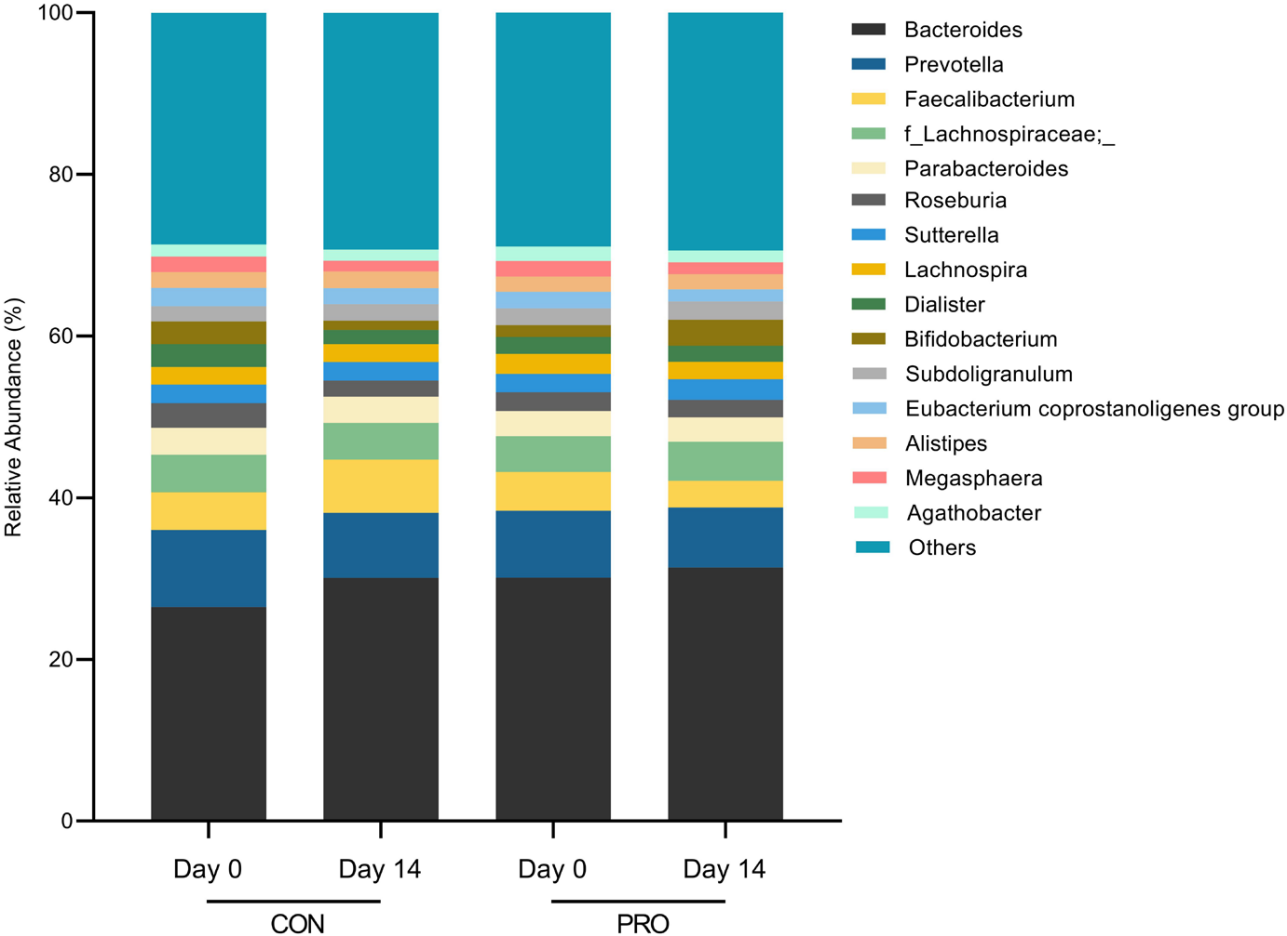
Relative abundance of gut microbiome at the genus level before and after each intervention period (*n* = 21).

The intestinal microbiota compositions of the 21 participants on a phylum and genus level are reflected in Figures 5 and 6, respectively. At a phylum level, gut microbiota predominantly comprised the *Bacteroidota* (43.9%), *Firmicutes* (45.6%), *Proteobacteria* (5.5%), and *Actinobacteriota* (3.3%). We have identified one phylum with significant differences to 14‐day changes in relative abundance between intervention groups. A significant increase in the relative abundance of *Proteobacteria* was observed on the 14th day of CON (7.44%, 95% CI [5.72, 9.16], *p* = 0.0247) from the 1st day of CON (5.52%, 95% CI [4.26, 6.79]), while no change in *Proteobacteria* abundance was observed after PRO.

At a genus level, gut microbiota predominantly comprised *Bacteroides* (26.5%). We have identified two genera with significant 14‐day changes in relative abundance between intervention groups. Relative abundance of *Bacteroides* increased on the 14th day of CON (30.1%, 95% CI [25.3, 34.9], *p* = 0.0429) from the 1st day of CON (26.5%, 95% CI [22.7, 30.2]). Also, the relative abundance of *Dialister* decreased on the 14th day of CON (1.77%, 95% CI [0.894, 2.66], *p* = 0.0411) relative to the 1st day of CON (2.85%, 95% CI [1.58, 4.13]). However, these changes were also not observed after PRO. In a secondary analysis of the changes in the relative abundance of the phylum *Proteobacteria*, and genera *Bacteroides* and *Dialister*, two‐way ANOVA did not show any significant time × intervention interactions (*Proteobacteria*: *F*(1, 20) = 2.17, *p* = 0.156; *Bacteroides*: *F*(1, 20) = 0.386, *p* = 0.541; *Dialister*: *F*(1, 20) = 2.86, *p* = 0.107) nor simple effects of time and intervention in both CON and PRO groups.

**FIGURE 7:**
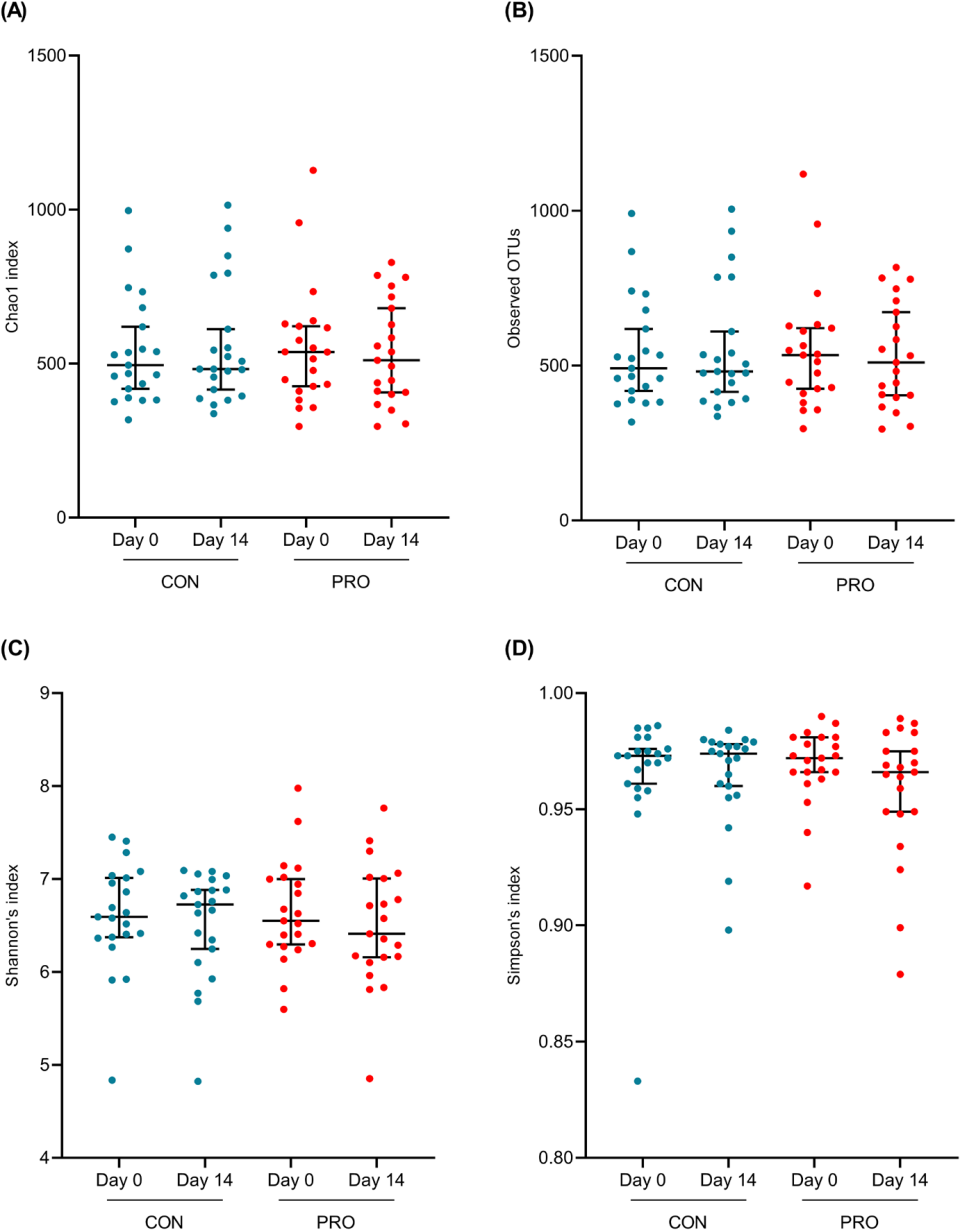
Differences in alpha diversity before and after the sour beer interventions (*n* = 21). (A) chao1 index, (B) observed OTUs, (C) Shannon's index, (D) Simpson's index. Graphs are presented as medians and 95% CI. Differences in postinterventional changes were analyzed with repeated measures one‐way ANOVA or Friedman test and statistical significance was determined at *p* < 0.05.

Alpha diversity indices of the 21 participants who had completed the study: chao1, observed operational taxonomic unit (OTU), Shannon's index, and Simpson's index are presented in Figure 7. Changes in alpha diversity indices over the 14‐day period did not reflect any statistically significant differences for either CON and PRO groups.

## 4 Discussion

The present study aimed to investigate the effects of a probiotic sour beer fermented with *L. paracasei* Lpc‐37 on the inflammatory, immunity, lipid profile, and gut microbiome of healthy male volunteers. Gut microbiome profiling indicated a significant increase in the phylum *Proteobacteria* and the genus *Bacteroides* and a significant decrease in the genus *Dialister* from baseline for CON. These changes were not observed for PRO. Alpha diversity indices also did not demonstrate any significant differences from baseline (chao1, observed OTUs, Shannon's index, and Simpson's index). On the other hand, a significant increase was observed in HDL‐c after PRO, but not after CON. No significant differences were observed in immunity WBCs (total count, neutrophils, lymphocytes, monocytes, eosinophils, and basophils) and for inflammatory markers (IFN‐γ, TNF‐α, and IL1b, 2, 4, 5, 10, 12p70).

Fundamentally, host health has been closely linked to the maintenance of a balanced gut microbial community (Liang, Saunders, and Sanossian 2023; Rinninella et al. 2019). Increasing evidence have demonstrated strong links between elevation of *Proteobacteria* abundance and gut dysbiosis, including being a microbial trademark of disease (Rizzatti et al. 2017; Shin, Whon, and Bae 2015). Several studies have also shown associations between an increase in *Proteobacteria* abundance in alcoholics as compared to nonalcoholics (Bjorkhaug et al. 2019; Mutlu et al. 2012; Queipo‐Ortuno et al. 2012), suggesting differences in microbial communities among these two groups of individuals. Additionally, *Proteobacteria* has also been found to be low in abundance in the gut microbiome of healthy individuals, and high among individuals in diseased conditions (Martinez‐Vega, Galvan‐Menendez‐Conde, and Freyre‐Fonseca 2023; Shin, Whon, and Bae 2015). A significant increase in *Bacteroides* has also been implicated in a distinct gut microbiome signature for alcoholic liver disease patients, together with genus *Akkermansia* (Addolorato et al. 2020), though the latter was not examined in the present study. A decrease in *Dialister* has also been linked to other diseased states (Caso et al. 2021; Gungor et al. 2016; Ramirez‐Carrillo et al. 2020).

Changes in these microbial communities at the phylum and genus level were observed in those following 2 weeks of daily CON consumption. Interestingly, these changes were not observed in the PRO group. Probiotics have also been reported to reverse alcohol‐induced changes in the microbiome for both human and mice (Fuenzalida et al. 2021; Kirpich et al. 2008; Wang et al. 2023), and the modulation of *L. paracasei* strains on the gut microbiome has been exhibited previously (Bengoa et al. 2021; Han et al. 2022). While its specific mechanism is still uncertain, probiotics such as *L. paracasei* have been suggested to modulate the gut microbiome through reduction in harmful bacteria (Di Cerbo et al. 2016; Gyawali et al. 2023), production of beneficial metabolites (Di Luccia et al. 2022), and improved intestinal barrier function (Fuenzalida et al. 2021; Li et al. 2021; Rupa and Mine 2012). Thus, *L. paracasei* Lpc‐37 fermented in PRO may have played a role in maintenance, restoration, and modulation of the gut microbiome.

The alpha diversity indices remained similar in both interventions, which signified that the 2‐week consumption of both PRO and CON beer had limited shifts in richness, evenness, and uniformity of gut microbial species, indicating stability in diversity after both interventions. While studies have reported alterations in gut microbial diversity following moderate alcohol consumption (Kosnicki et al. 2019; Lee and Lee 2021) and probiotic supplementation (Kato‐Kataoka et al. 2016; Plaza‐Diaz et al. 2015), the shorter 2‐week period for each intervention in the present study may not be a sufficient duration to observe any changes in gut microbial diversity. However, the presence of *L. paracasei* Lpc‐37 in the gut microbiome was confirmed through further sequencing analysis of the stool samples, which was found across all four time points (i.e., before and after PRO and CON), also indicating the presence of *L. paracasei* Lpc‐37 in the microbiome at baseline for some participants (Figure S2; Tables S1 and S2). *L. paracasei* Lpc‐37 was also detected for the largest proportion of participants (90%) after PRO, though this may not have been significant enough to influence any changes in the gut microbiome compositions or the alpha diversity indices.

The gut microbiome has also been reported to modulate blood lipid levels including HDL‐c levels (Fu et al. 2015; Ghanbari et al. 2024). Similarly, other studies have also demonstrated an increase in HDL‐c after supplementation with *Lacticaseibacillus* species probiotics in both healthy and diabetic individuals (Kocsis et al. 2020; Rajkumar et al. 2014; Sadrzadeh‐Yeganeh et al. 2010), though some studies revealed otherwise (Jones, Martoni, and Prakash 2012; Michael et al. 2020). However, the exact mechanisms underlying the interplay between the gut microbiome and blood lipid levels have not been fully established for elucidations to be made. Nevertheless, Wu et al. (2022) recently described that bile salt hydrolases (BSH) and SCFAs produced by probiotics may participate in lipid metabolism. *Lacticaseibacillus* strain *L. casei* LC2W was found to overexpress the BSH gene pWQH01, and another strain *L. plantarum* AR113 displayed increased BSH activity, both of which were shown to improve hepatic lipid buildup in a BSH‐dependent manner in vitro (Huang et al. 2020). SCFAs may also influence lipid metabolism (Cao et al. 2021; Yamasaki et al. 2020) through a number of mechanisms involving activation of the liver cyclic adenosine monophosphate (cAMP) pathway, which may suppress hepatic lipid production and the activation of G‐protein‐coupled receptor GPR43, leading to the inhibition of liver cholesterol synthesis (Wu et al. 2022). Therefore, the products of fermentation of *L. paracasei* Lpc‐37 in PRO may have participated in lipid metabolism resulting in the increase in HDL‐c, though more studies are still required to ascertain its specific mechanisms.

The gut microbiome has also been associated with changes in immunity. SCFAs produced may serve as potent immunomodulators by regulating the differentiation, recruitment, and activation of neutrophils, monocytes, and lymphocytes in the immune system (Correa‐Oliveira et al. 2016). This has mainly been attributed to their ability to influence the production of inflammatory cytokines through the inhibition of HDACs (Dalile et al. 2019; Vinolo et al. 2011). However, fasting levels of both cytokines and WBC counts did not appear to have any significant changes in both PRO and CON groups, indicating that both interventions had no effects on the inflammatory and immune markers of the participants.

While our study explores the use of a probiotic *Lacticaseibacillus* species as starter cultures in the production of sour beer, the use of a *Lacticaseibacillus* species itself is not new to the beer industry. Traditionally, sour beer has been produced through spontaneous fermentation involving microbial species which include yeast (e.g., *Saccharomyces*) and bacterial (e.g., *Lacticaseibacillus, Acetobacter*) species (Dysvik et al. 2020). Although sour beers generally do not have unique compound profiles (Edgar Herkenhoff, Brodel, and Frohme 2024), a distinguishing feature of sour beers compared with other beers (e.g., ales and lagers) is their high concentration of organic acids (Dysvik et al. 2020). In sour beers utilizing *Lacticaisebacillus* species, *Lacticaseibacillus* produces lactic acid that increases the organic acids present in the beer, elevating the intensity of the sour flavor profile including its sour aroma, acidic taste, and mouthfeel, which are desirable organoleptic characteristics of sour beers (Dysvik et al. 2020; Yan et al. 2024). *Lacticaseibacillus* species are also broadly tolerant to ethanol (Shane Gold et al. 1992), though their growth in beer may be hindered by the presence of hop's iso‐α‐acids (Dysvik et al. 2020) as they have antimicrobial properties. Therefore, most sour beers contain little hop content (Herkenhoff 2024). Similarly, in the present study, hops were omitted in the brewing process of the sour beers used—this may also have enabled the *L. paracasei* Lpc‐37 species to thrive in the sour beers and be maintained at favorable concentrations to confer benefits to the participants.

Previous studies have also explored the use of probiotic bacteria in the production of sour beer. Chan et al. (2019) developed a sour beer beverage utilizing probiotic strain *L. paracasei* L26 cofermented with *S. cerevisiae* S‐04 in unhopped wort, demonstrating remarkable growth and stability of *L. paracasei* L26 and significant concentrations of lactic acid production. Praia et al. (2022) also evaluated the feasibility of a sour beer with probiotic strain *L. paracasei* F19 with *S. cerevisiae* US‐05 using *Spondias mombin* L. juice and by‐product. Likewise, the authors reported lower viability of *L. paracasei* F19 in hopped wort, and also in bagasse or juice. Despite this, specific probiotic strains such as *L. paracasei* F19 and *L. paracasei* 431 used in the formulations of sour beers have been identified to be more tolerant and stable in elevated hop levels as compared to other strains, a characteristic that is attributed to higher expression levels of the bsrA gene (Herkenhoff 2024). *L. paracasei* F19 and *L. paracasei* 431 also have membrane adhesion proteins and H+ pumps, which may serve as defense mechanisms in beer brewing (Herkenhoff et al. 2023). These studies highlight promising prospects and feasibility of using beer as a delivery vehicle for probiotics, paving the way for a more healthful beer beverage alternative.

The strengths and limitations of the present study also need to be recognized. The randomized controlled, within‐subject crossover design of this study increases the statistical power of the study, and the free‐living conditions of participants allow for the assessment of PRO in a more realistic environment. However, the lack of a food diary during the intervention periods did not allow for tracking of the participants' dietary habits, which may influence the clinical parameters tested, especially for the gut microbiome. The 2‐week period for each intervention may also be an insufficient duration to observe the optimal effects of PRO, and the current results may also not be translatable for females as only males were recruited in this study. Future research may incorporate the above factors to further explore the clinical effects of PRO.

In conclusion, the 2‐week consumption of CON resulted in an increase in phylum *Proteobacteria* and genus *Bacteroides*, and a decrease in genus *Dialister*, while these alcohol‐induced changes were not observed for the PRO group. This indicated that PRO group did not result in any taxonomic gut microbial changes at the phylum and genus level. Both interventions also did not reflect changes in alpha diversity indices, which suggested stability in diversity and limited shifts in richness, evenness, and uniformity of gut microbial species. Additionally, a significant increase in HDL‐c levels was found in the PRO group but not for CON. Both PRO and CON groups also showed no effects on WBC counts and cytokines, suggesting minimal effects on immune and inflammatory markers. Overall, this study demonstrated PRO exhibiting limited effects on alcohol‐induced gut microbiome changes, alpha diversity, and immune and inflammatory markers. More research is required to ascertain its HDL‐c increasing potential and its link with the gut microbiome.

## Author Contributions

**Sean Jun Leong Ou:** formal analysis (equal), investigation (equal), project administration (equal), writing – original draft (equal). **Hafizah Yusri:** formal analysis (equal), investigation (equal), project administration (equal), writing – original draft (equal). **Dimeng Yang:** investigation (supporting), project administration (supporting). **Chin Meng Khoo:** resources (equal), supervision (supporting). **Mei Hui Liu:** conceptualization (lead), methodology (lead), resources (equal), supervision (lead), validation (lead), visualization (lead), writing – review and editing (lead).

## Ethics Statement

This study was approved by the National Healthcare Group Domain Specific Review Board (Ref 2021/00196).

## Consent

Written informed consent was obtained from all study participants.

## Conflicts of Interest

The authors declare no conflicts of interest.

## Supporting information


Appendix S1

## Data Availability

The data that support the findings of this study are openly available in the EBI repository at https://www.ebi.ac.uk/ena/browser/view/PRJEB70620, accession number PRJEB70620.
