# Supplementary material for: Effects of Moderate Consumption of a Probiotic‐Fermented Sour Beer on the Inflammatory, Immunity, Lipid Profile, and Gut Microbiome of Healthy Men in a Participant‐Blinded, Randomized‐Controlled Within‐Subject Crossover Study
Source: Food Sci Nutr. 2024 Dec 1;12(12):10867–80. doi: 10.1002/fsn3.4627 (PMC11666817; doi:10.1002/fsn3.4627)
Supplement: Supplementary file 1 — Appendix S1 [file FSN3-12-10867-s001.docx]

Supplementary Material

Effects of moderate consumption of a Probiotic-fermented Sour Beer on the inflammatory, immunity, lipid profile and gut microbiome of healthy men in a participant-blinded, randomized-controlled within-subject crossover study

Sean Jun Leong Ou^1†^, Hafizah Yusri^1†^, Dimeng Yang^1^, Chin Meng Khoo^2^, Mei Hui Liu^1*^

^1^Department of Food Science & Technology, National University of Singapore, Science Drive 2, 117542, Singapore

^2^Department of Medicine, Yong Loo Lin School of Medicine, National University of Singapore, 10 Medical Dr, 117597, Singapore

*** Correspondence:**Mei Hui Liu
Tel: +65 6516 3523
Email: fstlmh@nus.edu.sg

^†^Authors contributed equally to this work.

# Supplementary Material 1: Microbial enumeration of *Lacticaseibacillus* in PRO

The survivability of *Lacticaseibacillus* in PRO over a period of 264 days is presented in **Figure S1**. ‘Minimum’ represents the minimum value of 6.5 log CFU/mL, which is the recommended probiotic cell count required to attain health benefits. Cell viability of *Lacticaseibacillus* in PRO had decreased steadily over the 264-day storage period at 5^o^C and fell below the recommended limit of 6.5 log CFU/mL on day 264.


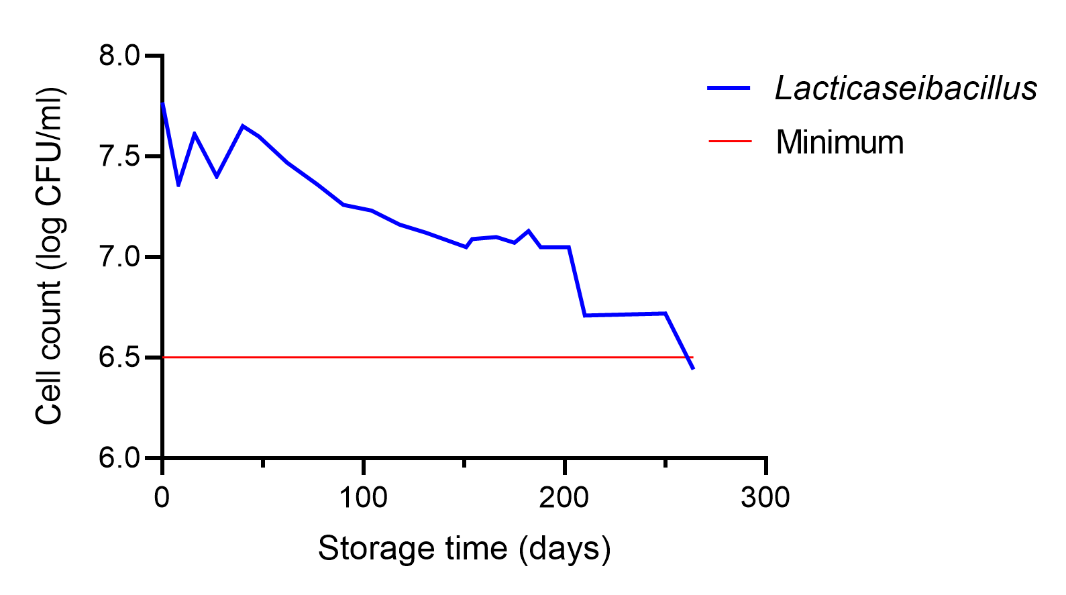


Figure S1. Cell count of *Lacticaseibacillus* in PRO beer stored at 5°C over a period of 264 days.

# Supplementary Material 2: Detection of Lpc-37 species in stool samples

Presence of Lpc-37 species in stool samples were confirmed by further sequencing. Lpc-37 was detected across all four timepoints for varying participant counts. **Figures S2** illustrates the number of participants with matched Lpc-37 sequences based on their order of interventions. Taken together, 55% (11/20) of participants had matched Lpc-37 sequences at baseline before study commencement. A total of 90% (18/20) of participants had matched Lpc-37 sequences after PRO, but only 65% (13/20) of participants had matched sequences after CON. Individual matched Lpc-37 sequences for each participant are displayed in **Tables S1** and **S2**.


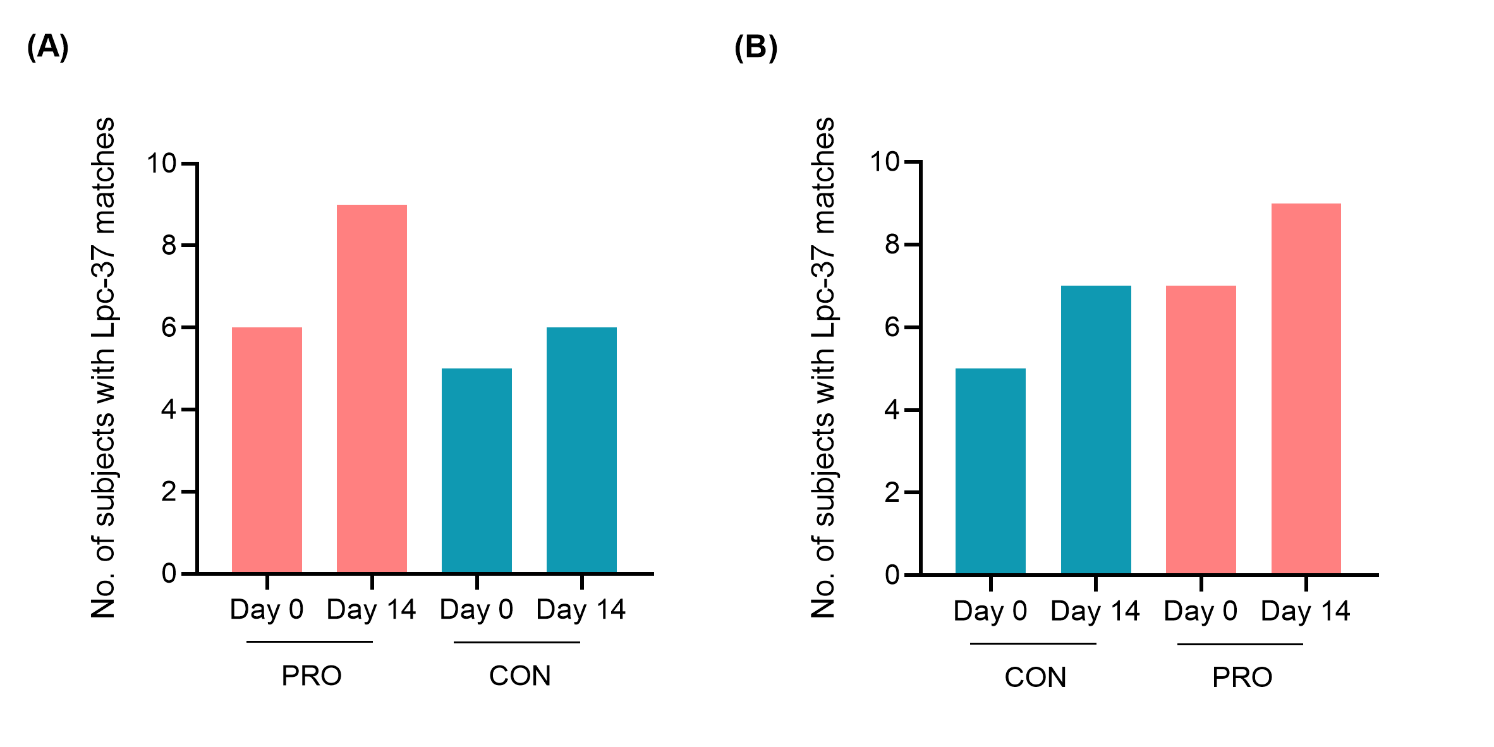


Figure S2. No. of participants with matched Lpc-37 sequences from BLAST for (A) participants following sequence of intervention starting with PRO first (n=10), and (B) participants following sequence of intervention starting with CON first (n=10).

**Table S1.** BLAST results of matched Lpc-37 sequences for participants with PRO as the first intervention (n=10).

| No. | PRO | | CON | |
| --- | --- | --- | --- | --- |
|  | Day 0 | Day 14 | Day 0 | Day 14 |
| 1 | X | ✓ | X | X |
| 2 | X | ✓ | ✓ | ✓ |
| 3 | ✓ | ✓ | ✓ | ✓ |
| 4 | X | ✓ | X | ✓ |
| 5 | ✓ | ✓ | X | ✓ |
| 6 | ✓ | ✓ | ✓ | ✓ |
| 7 | X | ✓ | ✓ | X |
| 8 | ✓ | ✓ | ✓ | ✓ |
| 9 | ✓ | ✓ | X | X |
| 10 | ✓ | X | X | X |

**Table S2.** BLAST results of matched Lpc-37 sequences for participants with CON as the first intervention (n=10).

| No. | CON | | PRO | |
| --- | --- | --- | --- | --- |
|  | Day 0 | Day 14 | Day 0 | Day 14 |
| 1 | X | X | ✓ | ✓ |
| 2 | X | ✓ | ✓ | ✓ |
| 3 | ✓ | ✓ | ✓ | ✓ |
| 4 | X | ✓ | ✓ | ✓ |
| 5 | ✓ | ✓ | ✓ | ✓ |
| 6 | ✓ | ✓ | ✓ | ✓ |
| 7 | ✓ | ✓ | ✓ | ✓ |
| 8 | ✓ | X | X | ✓ |
| 9 | X | X | X | ✓ |
| 10 | X | ✓ | X | X |
